# Steadfast Toll Like Receptor 4 (*TLR4*) 5-Hydroxymethylcytosine Levels in Cell-Free DNA: A Promising Consistency Marker for Colorectal Cancer Patients

**DOI:** 10.3390/genes14081636

**Published:** 2023-08-17

**Authors:** Daša Jevšinek Skok, Nina Hauptman

**Affiliations:** 1 Agricultural Institute of Slovenia, Hacquetova ulica 17, SI-1000 Ljubljana, Slovenia; dasa.jevsinekskok@kis.si; 2 Institute of Pathology, Faculty of Medicine, University of Ljubljana, Korytkova 2, SI-1000 Ljubljana, Slovenia

**Keywords:** cell-free DNA, colon cancer, 5-hydroxymethylcytosine, biomarkers

## Abstract

Cell-free DNA (cfDNA) from patient blood is emerging as a noninvasive diagnostic avenue for various cancers. We aimed to identify reliable biomarkers in cfDNA by investigating genes exhibiting significant differences between colorectal cancer and control samples. Our objective was to identify genes that showed a positive difference between cancer and control samples. To achieve this, we conducted an in silico analysis to identify genes that exhibit no significant variation in methylation between genomic DNA (gDNA) and cfDNA. We collected experimental data from publicly available repositories, which included 5-hydroxymethylcytosine (5hmC) profiles of gDNA and cfDNA samples from both cancer patients and healthy individuals. By comparing and overlapping these two groups, we identified 187 genes of interest, of which 53 genes had a positive difference among colon cancer patients and healthy individuals. Next, we performed an ANOVA test on these genes, resulting in the identification of 12 genes that showed statistically significant higher levels of 5hmC in cfDNA and gDNA from cancer patients compared to healthy individuals. Additionally, we compared the 5hmC status of these genes between cfDNA and gDNA from cancer patients. Interestingly, we found that the 5hmC of the toll like receptor 4 (*TLR4*) gene was not statistically different between cfDNA and gDNA from cancer patients, indicating consistency between cfDNA and gDNA. These findings have important implications, not only for experimental validation but also for the development of more sensitive and robust noninvasive methods to improve diagnostic, prognostic, and treatment options for colon cancer.

## 1. Introduction

Transcriptional regulation encompasses a wide array of mechanisms, with epigenetic modifications playing a crucial role in gene expression control. Among these modifications, DNA methylation and histone modifications are particularly significant. DNA methylation is involved in numerous biological processes, including embryonic development, genomic imprinting, X-chromosome inactivation, cellular differentiation, and cancer development [1]. The primary form of DNA methylation involves the addition of a methyl group to the C5 position of a cytosine residue, catalyzed by DNA methyltransferases, leading to the formation of 5-methylcytosine (5mC). Notably, the demethylation process is facilitated by the ten–eleven translocation (TET) family of cytosine oxygenases. Through oxidation, 5mC can be converted into an intermediate state known as 5-hydroxymethylcytosine (5hmC) [1]. The presence of 5hmC has a critical role not only as an intermediate in the active DNA demethylation process during early developmental stages but also as a persistent and stable epigenetic modification following epigenetic reprogramming. Extensive research has demonstrated the detectability of 5hmC in diverse tissues and cell types, exhibiting varying levels of this epigenetic mark [2,3], including colorectal cancer [4].

Colorectal cancer (CRC) is one of the most common cancers and is one of the leading causes of death worldwide [5]. The current methods for CRC screening are predominantly invasive [6], with colonoscopy as a widely used method for the examination of the entire colon and the removal of lesions. On the other hand, screening tests to detect CRC, such as the fecal occult blood test, stool DNA, sigmoidoscopy, colonoscopy, and CT colonography, have been shown to be an effective way to identify early CRC and precancerous lesions, thus reducing disease morbidity and mortality [7]. Blood-based biomarkers are capable of improving CRC screening adherence, and a large number of candidate biomarkers have been reported for CRC diagnosis, as reviewed in [8]. One of the noninvasive biomarkers is circulating cell-free DNA (cfDNA), which is a promising biomarker for several diseases, including cancer [9,10]. cfDNA are fragments of DNA found in the blood that are the result of apoptosis in different tissues and can be used for the noninvasive detection of cancer.

Recently, the epigenetic state of 5hmC was discovered to be involved in various biological processes, including pathogenesis. Low levels of 5hmC were observed in many solid tumors compared to normal tissues, suggesting their potential usefulness in cancer diagnostics [8,11]. The levels of 5hmC in tissue do not necessarily mean high levels of 5hmC in cfDNA since the cfDNA in the blood is not protected and is exposed to different molecules and influences. Aberrant DNA methylation detected in liquid biopsies, such as serum circulating cfDNA, is a promising source of non-invasive biomarkers. Therefore, there is an imperative need to find new non-invasive biomarkers for CRC screening.

In this study, we focused on determining genes that exhibited a positive difference between cancer and controls. Furthermore, we aimed to find genes where the levels of 5hmC in cfDNA and tissue were comparable, and there was little difference between them. These genes would be good candidate biomarkers due to their consistent level between cfDNA and genomic DNA (gDNA).

## 2. Materials and Methods

We conducted an analysis using publicly available data from the Gene Expression Omnibus (GEO). The data were obtained with next-generation sequencing (NGS), focusing on 5hmC cfDNA from the blood and 5hmC gDNA from tissue samples. This study involved patients with colon cancer as well as healthy individuals.

Two projects were used in our analysis. The first project, GSE81314 [8] (accessed on 23 May 2023), employed the Illumina NextSeq 500 platform (San Diego, CA, USA) and included cfDNA samples from patients with colon cancer and healthy individuals. The second project, GSE89570 [11] (accessed on 23 May 2023), utilized the Illumina HiSeq 2000 platform. This project provided sequencing data for cfDNA from colon cancer patients and healthy individuals and sequencing data from the gDNA of colon cancer tissue and adjacent normal mucosa samples.

Specifically, we selected samples that were either colon cancer or healthy/normal. From project GSE81314, we included 4 cfDNA samples from colon cancer patients and 8 cfDNA samples from healthy individuals. From project GSE89570, we included 44 gDNA samples from colon cancer tissue, 44 gDNA samples from adjacent normal mucosa, 78 cfDNA samples from colon cancer patients, and 96 cfDNA samples from healthy patients.

To compare these groups, we employed the limma package (version 3.57.4) in R [12], performing pairwise comparisons between cfDNA samples from colon cancer patients and healthy individuals in each project, as well as between gDNA samples from colon cancer tissue and adjacent normal mucosa samples. For each gene, a false discovery rate (FDR) was determined, and the genes exhibiting adjusted *p*-values less than 0.05 in each comparison were used for further analysis. In the next step, we identified genes where the differences in levels of 5hmC between the cfDNA of colon cancer patients and cfDNA of healthy individuals were positive, and at the same time, the levels of 5hmC between the gDNA of colon cancer tissue and gDNA of healthy individuals adjacent to normal mucosa samples also was positive. These genes were further subjected to an ANOVA test with Bonferroni post hoc analysis using IBM SPSS Statistics (version 24, IBM., Armonk, New York, NY, USA).

Furthermore, the graphical representation of NGS results was generated for 12 genes using the R programming language (version 4.3.0) [12]. The creation of these graphs was facilitated by the utilization of the ggplot2 library (version 3.4.2) [13] with specific emphasis on the geom_boxplot function. Boxplots, a type of data visualization, were employed to illustrate summary statistics for our dataset, encompassing what is commonly referred to as the “five number summary”. This summary encompasses essential statistical values, including the minimum, first quartile (25th percentile), median, third quartile (75th percentile), and maximum.

## 3. Results

**Figure 1:**
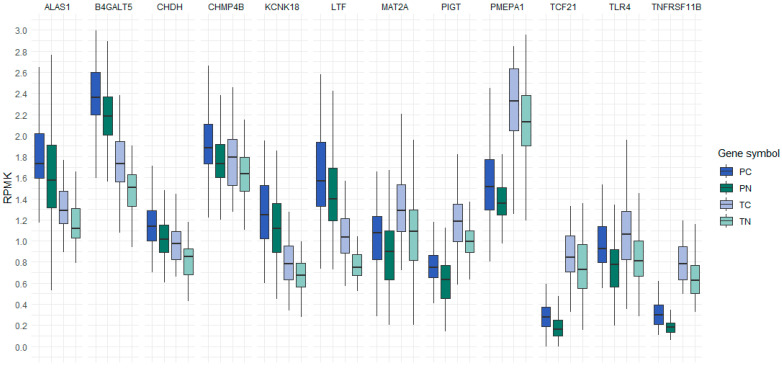
Reads per million kilobases of 12 genes with elevated 5hmC levels in cfDNA of colon cancer patients compared to cfDNA of healthy individuals and elevated 5hmC levels in the gDNA of colon cancer tissue samples compared to the gDNA of adjacent normal tissue samples. The boxplot compactly displays the distribution of a continuous variable. It visualizes five summary statistics (the median, two hinges and two whiskers). RPKM, reads per million kilobases; PC, cfDNA from the plasma of colon cancer patients; PN, cfDNA from the plasma of healthy individuals; TC, gDNA from colon cancer tissue samples; TN, gDNA from adjacent normal tissue samples.

Pairwise comparisons within each project between colon cancer cfDNA and healthy individuals cfDNA or colon cancer and adjacent normal tissue samples revealed 187 genes with an adjusted *p*-value less than 0.05. Among these, 53 genes displayed a positive difference in their 5hmC levels in the colon cancer cfDNA or gDNA compared to healthy plasma or adjacent normal tissue samples. Further analysis, using ANOVA with Bonferroni post hoc testing, identified 12 genes with elevated 5hmC levels in the cfDNA of colon cancer patients compared to healthy individuals in both GEO projects. These same 12 genes were also found with increased levels of 5hmC in gDNA from cancer tissues compared to adjacent normal tissues. These genes were: 5’-aminolevulinate synthase 1 (*ALAS1*), beta-1,4-galactosyltransferase 5 (*B4GALT5*), choline dehydrogenase (*CHDH*), charged multivesicular body protein 4B (*CHMP4B*), potassium two pore domain channel subfamily K member 18 (*KCNK18*), lactotransferrin (*LTF*), methionine adenosyltransferase 2A (*MAT2A*), phosphatidylinositol glycan anchor biosynthesis class T (*PIGT*), prostate transmembrane protein, androgen induced 1 (*PMEPA1*), transcription factor 21 (*TCF21*), toll like receptor 4 (*TLR4*), and TNF receptor superfamily member 11b (*TNFRSF11B*). These results are depicted in Figure 1, where 5hmC levels are presented as reads per million kilobases (RPKM).

The pairwise comparison test between cfDNA from colon cancer patients compared to the cfDNA of healthy individuals and between the gDNA of colon cancer tissue samples and gDNA of adjacent normal samples revealed that the 5hmC status was significantly different in all 12 genes (adjP < 0.05) (Table 1). It is interesting that *TLR4* is the only gene where there was no significant difference between levels of cfDNA from colon cancer patients and gDNA from colon cancer tissue samples (adjP > 0.05). The second gene where the adjusted *p*-value between the cfDNA of colon cancer patients and the gDNA of colon cancer tissue samples was somewhat higher was *CHMP4B* (adjP = 0.02). The highest level of 5hmC in the cfDNA of colon cancer patients was observed in gene *B4GALT5* with an estimated mean value of 2.51 RPKM which is 0.30 RPKM higher than in the cfDNA of healthy patients. Significantly higher levels of 5hmC of gene *B4GALT5* were also observed in the gDNA of colon cancer tissue samples versus adjacent normal samples, where the estimated value of colon cancer tissue was 1.80 RPKM; 0.32 RPKM higher than in the gDNA of normal adjacent tissue. The highest significant difference between colon cancer and adjacent normal tissue was observed for gene *PMEPA1* with an estimated mean value of 3.01 RPKM in gDNA colon cancer tissue samples; 0.72 RPKM higher than in adjacent normal tissue. Moreover, the 5hmC level of gene *PMEPA1* was 0.35 RPKM higher in the cfDNA of colon cancer patients than in healthy individuals.

## 4. Discussion

In this study, we analyzed the available data of 5hmC levels of cfDNA from colon cancer and cfDNA from healthy individuals and gDNA from colon cancer tissue, and gDNA from adjacent normal tissue samples from GEO projects. The analysis revealed 12 genes with increased levels of 5hmC in cfDNA and the gDNA of colon cancer patients compared to healthy individuals/normal adjacent samples in all GEO projects. From those genes, *TLR4* is the only gene where there was no significant difference between the levels of 5hmC in colon cancer cfDNA and colon cancer gDNA.

Toll-like receptors (TLRs), spanning TLR1 through TLR10, are members of a highly conserved molecule class. Their predominant presence in immune cells facilitates the recognition of pathogen-associated molecular patterns or damage-associated molecular patterns [14,15]. Emerging investigations also unveil the potential antitumor impacts associated with TLRs [16]. This innate immune cell response involves cytokine secretion, enabling cancer cell elimination and the recruitment of adaptive immune cells by activating TLR signaling pathways [17,18]. Through direct engagement with TLRs on tumor cells, TLR agonists have been observed to induce apoptosis, inhibit proliferation, and curtail migration [19,20]. Notably, certain TLR agonists have found an application in clinical contexts, as exemplified by Bacille Calmette-Guérin (BCG), a triagonist of TLR2, TLR4, and TLR9, in preventing non-muscle invasive bladder cancer recurrence [21]. The precise mechanism driving TLRs’ impact on the tumor immune microenvironment remains enigmatic. Previous research has reported the expression profile of TLR genes, with a focus on TLR4, within bladder cancer, with changes in promoter methylation levels of TLR4, shedding light on alterations in infiltrating immune cells and uncovering shifts in functional pathways [14].

In our study, we identified 12 genes, among which gene *B4GALT5* exhibited the highest levels of 5hmC in colon cancer cfDNA. *B4GALT5* encodes type II membrane-bound glycoproteins that function as enzymes for lactosylceramide and glycosaminoglycan chain biosynthesis. Previous research has shown that *B4GALT5* is regulated by Ets family transcription factors, including Ets-1 and Ets-2, in cancer cells [22,23,24]. However, the biological functions of *B4GALT5* in CRC are poorly understood.

We observed *PMEPA1* with the highest difference in 5hmC status between cancer and normal tissue. *PMEPA1* is highly expressed in prostate epithelial cells [25] and is methylated in prostate cancer [26]. Moreover, its methylation and mRNA expression in the same tumor of cell populations indicated a significant inverse correlation between mRNA expression and methylation status. 

Little is known about the effect of our identified 12 genes, especially on CRC; therefore, this study has important clinical potential to provide efficient targets for the early diagnostics of CRC. However, further experimental validation is needed to confirm the action of developing new diagnostic markers for CRC.

## Figures and Tables

**Table 1:** Descriptive statistics and differences in 5hmC status for 12 genes in cfDNA from colon cancer patients and healthy individuals and in colon cancer tissue samples and adjacent normal samples.

Gene	Estimated Marginal Means				Pairwise Comparison for					
	PC	PN	TC	TN	PC-PN	TC-TN	PC-TC	Adjusted *p*-Value		
								PC-PN	TC-TN	PC-TC
*ALAS1*	1.81	1.62	1.39	1.18	0.19	0.21	0.42	6.34 × 10^−7^	5.09 × 10^−4^	6.55 × 10^−19^
*B4GALT5*	2.51	2.21	1.80	1.48	0.30	0.32	0.71	8.24 × 10^−15^	1.63 × 10^−8^	4.88 × 10^−52^
*CHDH*	1.16	1.04	0.98	0.81	0.11	0.17	0.17	1.65 × 10^−4^	6.41 × 10^−3^	1.30 × 10^−3^
*CHMP4B*	1.95	1.74	1.79	1.62	0.21	0.17	0.16	5.51 × 10^−11^	6.35 × 10^−3^	4.58 × 10^−3^
*KCNK18*	1.32	1.14	0.86	0.70	0.18	0.16	0.46	2.90 × 10^−8^	1.52 × 10^−2^	4.56 × 10^−22^
*LTF*	1.71	1.49	1.16	0.77	0.23	0.40	0.55	1.03 × 10^−15^	4.96 × 10^−13^	9.30 × 10^−32^
*MAT2A*	1.05	0.90	1.38	1.07	0.15	0.31	−0.33	3.77 × 10^−1^	2.73 × 10^−8^	1.00 × 10^−11^
*PIGT*	0.81	0.64	1.19	0.99	0.17	0.20	−0.38	4.13 × 10^−3^	7.05 × 10^−4^	7.02 × 10^−16^
*PMEPA1*	1.69	1.35	3.01	2.30	0.35	0.72	−1.32	4.64 × 10^−22^	5.57 × 10^−41^	6.51 × 10^−172^
*TCF21*	0.31	0.19	0.93	0.75	0.12	0.18	−0.62	1.47 × 10^−1^	3.49 × 10^−3^	1.92 × 10^−39^
*TLR4*	0.98	0.79	1.07	0.81	0.19	0.26	−0.09	1.07 × 10^−3^	7.86 × 10^−6^	3.61 × 10^−1^
*TNFRSF11B*	0.33	0.19	0.83	0.64	0.14	0.19	−0.50	1.38 × 10^−3^	2.71 × 10^−3^	8.79 × 10^−26^

PC, cfDNA from plasma of colon cancer patients; PN, cfDNA from plasma of healthy individuals; TC, gDNA from colon cancer tissue samples; TN, gDNA from adjacent normal tissue samples.

## Data Availability

All data used in this paper are available in the article.
